# High expression of Trop2 is associated with aggressive localized prostate cancer and is a candidate urinary biomarker

**DOI:** 10.1038/s41598-023-50215-z

**Published:** 2024-01-04

**Authors:** Shiqin Liu, Sarah J. Hawley, Christian A. Kunder, En-Chi Hsu, Michelle Shen, Lennart Westphalen, Heidi Auman, Lisa F. Newcomb, Daniel W. Lin, Peter S. Nelson, Ziding Feng, Maria S. Tretiakova, Lawrence D. True, Funda Vakar-Lopez, Peter R. Carroll, Jeffry Simko, Martin E. Gleave, Dean A. Troyer, Jesse K. McKenney, James D. Brooks, Michael A. Liss, Tanya Stoyanova

**Affiliations:** 1 Department of Molecular and Medical Pharmacology, University of California Los Angeles, Los Angeles, CA USA; 2 Canary Foundation, Woodside, CA USA; 3 Department of Pathology, Stanford University, Palo Alto, CA USA; 4 Department of Radiology, Stanford University, Palo Alto, CA USA; 5 Department of Urology, University of Washington, Seattle, WA USA; 6 Division of Human Biology, Fred Hutchinson Cancer Center, Seattle, WA USA; 7 Program of Biostatistics and Biomathematics, Division of Public Health Sciences, Fred Hutchinson Cancer Center, Seattle, WA USA; 8 Department of Laboratory Medicine and Pathology, University of Washington Medical Center, Seattle, WA USA; 9 Department of Urology, Helen Diller Family Comprehensive Cancer Center, University of California San Francisco, San Francisco, CA USA; 10 Department of Urologic Sciences, University of British Columbia, Vancouver, BC Canada; 11 Department of Pathology, Eastern Virginia Medical School, Norfolk, VA USA; 12 Department of Anatomic Pathology, Cleveland Clinic, Cleveland, OH USA; 13 Department of Urology, Stanford University, Palo Alto, CA USA; 14 Department of Urology, University of Texas Health Science Center at San Antonio, San Antonio, TX USA; 15 Department of Urology, University of California, Los Angeles, Los Angeles, CA USA

**Keywords:** Tumour biomarkers, Urological cancer

## Abstract

Distinguishing indolent from clinically significant localized prostate cancer is a major clinical challenge and influences clinical decision-making between treatment and active surveillance. The development of novel predictive biomarkers will help with risk stratification, and clinical decision-making, leading to a decrease in over or under-treatment of patients with prostate cancer. Here, we report that Trop2 is a prognostic tissue biomarker for clinically significant prostate cancer by utilizing the Canary Prostate Cancer Tissue Microarray (CPCTA) cohort composed of over 1100 patients from a multi-institutional study. We demonstrate that elevated Trop2 expression is correlated with worse clinical features including Gleason score, age, and pre-operative PSA levels. More importantly, we demonstrate that elevated Trop2 expression at radical prostatectomy predicts worse overall survival in men undergoing radical prostatectomy. Additionally, we detect shed Trop2 in urine from men with clinically significant prostate cancer. Our study identifies Trop2 as a novel tissue prognostic biomarker and a candidate non-invasive marker for prostate cancer.

## Introduction

Prostate cancer is the most common non-cutaneous cancer among men in the United States, accounting for about 29% of cancer diagnoses^1^. It is projected that 34,700 men will die from prostate cancer in 2023, making it the second leading cause of cancer-related deaths among men in the United States^1^. Current screening approaches for prostate cancer center on the measurement of serum prostate-specific antigen (PSA) in combination with adjuncts such as measurement of molecular forms of PSA, multiparametric MRI, and digital rectal examination^2–6^. Clinically significant prostate cancer often fails local therapies, such as surgery or radiation therapy, and often leads to metastatic disease and mortality^7–9^. Stratifying clinically significant prostate cancer from indolent prostate cancer using clinical features alone such as PSA level, Gleason Score, or T-stage is often challenging, particularly for intermediate-risk disease^3,7,9–11^. The development of new molecular prognostic biomarkers could help with risk stratification and clinical decision-making by better identification of clinically significant prostate cancer^12,13^.

Trophoblastic cell surface antigen-2 (Trop2), an oncogenic transmembrane cell surface protein, is highly expressed in metastatic prostate cancer^14,15^. Trop2 defines a subpopulation of prostate basal cells that have self-renewal activity^16^. Trop2 promotes tumorigenicity, metastasis, and neuroendocrine phenotype in prostate cancer^15–17^. In our previous study, we demonstrated that high levels of tissue Trop2 predict a shorter time to recurrence in a prostate cancer cohort with 234 patients, suggesting that Trop2 could serve as a prognostic tissue biomarker for early detection of clinically significant localized prostate cancer^15^. Additionally, Trop2 is cleaved via a protease, disintegrin and metalloproteinase 17 (ADAM17), resulting in the release of the Trop2 extracellular domain into the extracellular environment^18^, making it a potential liquid marker of prostate cancer.

In this study, we validate the prognostic power of Trop2 expression in prostate cancer tissue utilizing the Canary Prostate Cancer Tissue Microarray (CPCTA) which contains over 1100 patient samples, and assess urine shed Trop2 as a potential non-invasive biomarker for diagnosis and prediction of clinically significant prostate cancer. We demonstrate that elevated levels of Trop2 significantly correlate with shorter overall survival, higher Gleason Score, and higher pre-operative PSA levels. In addition, shed Trop2 can be detected in the urine from men with clinically significant prostate cancer. These findings suggest that Trop2 holds promise to serve as a novel prognostic tissue biomarker and diagnostic urine marker for prostate cancer that could be used to optimize treatment decision-making.

## Results

### Positive Trop2 expression is associated with worse overall survival

**Figure 1:**
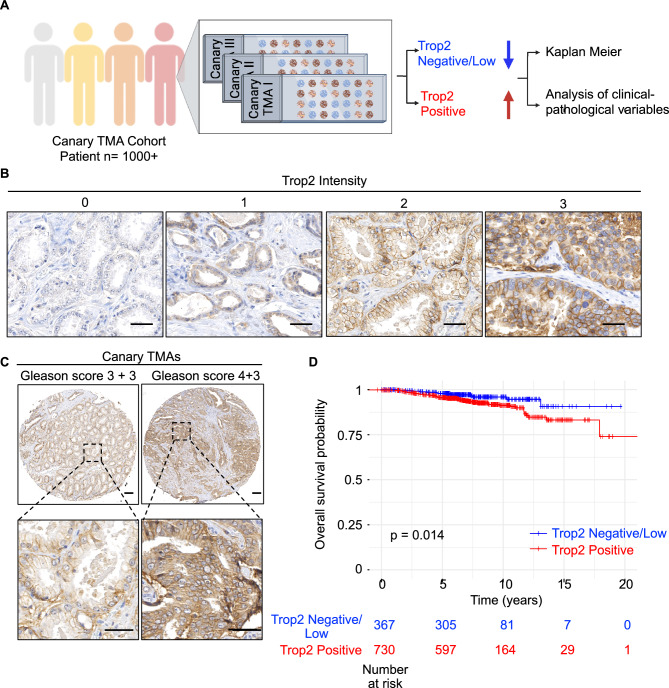
Positive Trop2 expression is associated with worse overall survival time. (**A**) Schematic representation of experimental design. The image was generated using BioRender (https://biorender.com). (**B**) IHC staining of Trop2 in the Canary Prostate Cancer Tissue Microarray (CPCTA) cohort (n = 1153). Trop2 staining was subject to scoring from 0 to 3. 0 = no staining; 1 = not interpretable/core missing; 2 = faint staining; 3 = dense staining. The Canary TMAs included 4 cores/case. Trop2 expression is summarized as (1) positive in cases with at least one core with a score of 3 and (2) low/negative in cases where all cores have scores of 2 or less. Cases with no cancer found in any cores are excluded from the analysis. Scale bar = 40 microns. (**C**) Representative images of Trop2 staining in cancer cases from Canary TMAs. Scale bar = 100 microns (upper) and 40 microns (bottom). (**D**) Kaplan–Meier survival curves of overall survival in Canary TMA cohort. Trop2 positive predicted worse overall survival of prostate cancer patients at radical prostatectomy. n = 1097. Patients without survival time were excluded from the analysis. p = 0.014, by log-rank test.

To validate Trop2 as a prognostic tissue biomarker for clinically significant prostate cancer, we assessed tissue levels of Trop2 in the Canary Prostate Cancer Tissue Microarray (CPCTA) that was constructed from prostate cancer tissues from 1328 patients from 7 institutions with a minimum of 5 years follow-up (Fig. 1A)^19^. Cores from 1153 patients (87%) had sufficient tissue to evaluate Trop2 levels by immunochemistry (IHC). The pathologists scored the intensity of the Trop2 IHC staining from 0 to 3 without knowledge of sample metadata (Fig. 1B). Samples with at least one core (1 out of 4 cores) scored as 3 were categorized as positive Trop2 expression. Samples were categorized as low/negative Trop2 expression when all evaluable cores were scored as 2 or below. We found that 764 (66%) cases were Trop2 positive, and 389 (34%) cases were Trop2 low/negative (Table. 1).

**Table 1:** Summary of association between Trop 2 staining and clinical-pathologic variables at radical prostatectomy.

Variable	Trop 2 Negative (N = 389)	Trop 2 Positive (N = 764)	p-value
Gleason score			
≤ 6	222 (56%)	326 (43%)	
7	122 (32%)	327 (43%)	
≥ 8	45 (12%)	111 (14%)	< 0.001^&^
P-stage			
T2	256 (70%)	491 (66%)	
T3/T4	110 (30%)	258 (34%)	0.163^&^
Age (years)			
Median	62	63	0.023^+^
Pre-op PSA (ng/mL)			
Median	6.48	6.81	0.017^+^
Surgical margins			
Negative	259 (71%)	471 (65%)	0.052^&^
Positive	107 (29%)	257 (35%)	
ECE			
No	267 (79%)	511 (68%)	0.489^&^
Yes	114 (30%)	242 (32%)	
Seminal vesicle invasion			
No	358 (93%)	706 (94%)	0.710^&^
Yes	27 (7%)	47 (6%)	

^+^Wilcoxon Test; ^&^Pearson Chi-Squared Test.

We first analyzed the association between Trop2 expression and clinical-pathologic variables at radical prostatectomy. Trop2 positive expression correlated with unfavorable clinicopathological features including higher median age compared to Trop2 low/negative group (63 v. 62 years; p = 0.023, Wilcoxon Test), higher median log pre-operative PSA (1.92 v. 1.87; p = 0.017, Wilcoxon Test), and a greater likelihood of Gleason Score ≥ 7 in their radical prostatectomy specimen (57% v. 44%; p < 0.001, Pearson Chi-Squared Test) (Table 1, and Fig. 1C). There was no difference in the percentage of patients with positive surgical margins, extracapsular extension (ECE), seminal vesicle invasion (SVI), or P-stage T3/T4 between Trop2 high and Trop2 low/negative expression (Table 1). Importantly, Trop2 positive expression is associated with a shorter overall survival time in Kaplan–Meier analysis (p = 0.014) (Fig. 1D). Trop2 positive expression has a trend with shorter disease specific survival but did not achieve significance due to few prostate cancer deaths in the cohort. In univariate Cox proportional hazards analysis, patients with Trop2 positive expression had lower overall survival compared to those with Trop2 low/negative expression (Hazard Ratio (HR) 2.07; 95% Confidence Interval (CI) 1.13, 3.29, p = 0.018) (Table 2). Multivariate Cox proportional hazards analysis revealed that high Trop2 trended toward significant association with worse overall survival when adjusted for Gleason Score and age (Hazard Ratio (HR) 1.78; 95% Confidence Interval (CI) 0.96, 3.28, p = 0.066) (Table 2). Only Gleason Score and age were significant predictors of overall survival when all clinical and pathological features are included in a multivariate model. Together, these data support the hypothesis that elevated Trop2 levels at the time of radical prostatectomy for localized disease can serve as a tissue marker for aggressive prostate cancer.

**Table 2:** Cox proportional hazards models.

Event	Variable	Comparison	Hazard Ratio (95% CI)	P-value
UV: OS (N = 1100; 68 events, 1032 censored)	Trop2	At least one core > 2 (pos.) v. All cores ≤ 2 (neg.)	2.07 (1.13, 3.79)	0.018*
MV: OS (N = 997; 67 events, 930 censored)	Trop2	At least one core > 2 (pos.) v. All cores ≤ 2 (neg.)	1.78 (0.96, 3.28)	0.066**
	Gleason score 7	Gleason score 7 v. Gleason Score 6	1.18 (0.66, 2.10)	0.579
	Gleason score ≥ 8	Gleason score ≥ 8 v. ^Gleason^Score ≤ 6	2.49 (1.34, 4.61)	0.004
	Age	1-year change	1.06 (1.02, 1.11)	0.002*

*UV* univariate, *MV* multivariate.

*Statistically significant, p-value < 0.05.

**Borderline statistically significant, p-value < 0.10.

### Trop2 can be detected in urine from clinically significant prostate cancer patients

**Figure 2:**
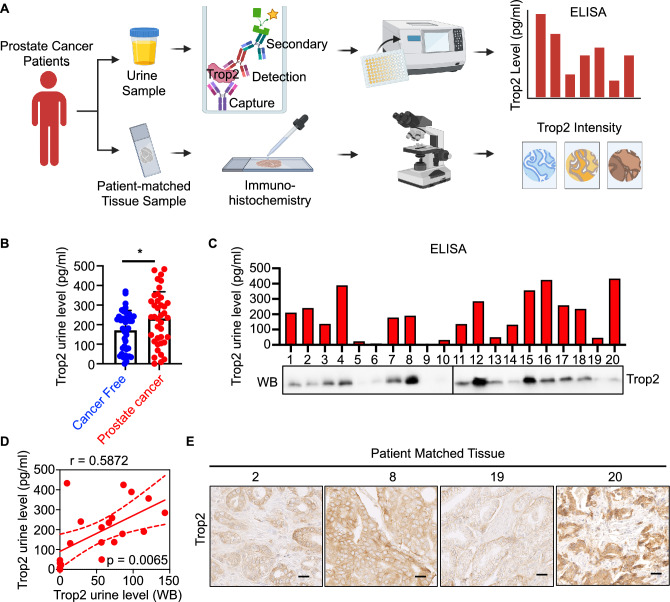
Trop2 can be detected in urine from clinically significant prostate cancer patients. (**A**) Schematic representation of experimental design (generated via BioRender (https://biorender.com). (**B**) Trop2 levels were assessed by ELISA in urine from cancer-free patients (n = 40) and prostate cancer patients (n = 39). p = 0.0285, determined by Student-T test (Two-tailed). (**C**) Trop2 levels in urine samples from patients with clinically significant prostate cancer were evaluated by ELISA (20ul/sample) or Western blot (30 μl urine/sample). For Western Blot, 10 patients were run in the same gel with positive control, and two blots were developed at the same time with the same exposure time. (**D**) The correlation of Trop2 levels from ELISA and WB analysis. P = 0.0065. (**E**) Trop2 levels in patient-matched tissues were evaluated by IHC staining. Scale bar = 25 microns.

Trop2 is cleaved and released into the extracellular environment, making it a potential liquid biomarker for prostate cancer^18^. Given our finding that elevated tissue levels of Trop2 were associated with poorer clinical features, we assessed whether shed Trop2 could be detected in the urine from men with clinically significant prostate cancer, thereby providing a non-invasive biomarker to identify clinically significant prostate cancer and monitor the disease. To test urine Trop2 levels, we developed a plate Sandwich enzyme-linked immunoassay (ELISA) by using two commercially available anti-Trop2 capture and detection antibodies (Fig. 2A). Urine samples from 40 patients with low serum PSA for 10 years were used as cancer-free controls (Supplementary Table 1). We obtained urine samples from 39 patients with clinically significant prostate cancer to test urine Trop2 levels. Approximately 69% of patients were defined as unfavorable intermediate-risk or above according to NCCN stratification (Supplementary Table 2). Compared to cancer free patients, shed Trop2 was detectable and was significantly higher in the urine from clinically significant prostate cancer patients (p = 0.0191, Fig. 2B). Western Blot analysis of the urine samples showed detection of a single band at the expected size, confirming the validity of the ELISA (Fig. 2C,D, and Supplementary Fig. 1). Our Trop2 ELISA assay represents a specific, high-throughput, and inexpensive method to detect shed Trop2 levels in urine (p = 0.0065) (Fig. 2C,D). We confirmed the expression of Trop2 protein in patient-matched tissues by IHC staining, and high levels of Trop2 were found in the cancer samples in all cases where shed Trop2 was found in the urine by ELISA (Fig. 2E). In summary, shed Trop2 could be detected in urine samples from clinically significant prostate cancer patients, suggesting that it could be used as a non-invasive marker for early detection of clinically significant prostate cancer.

## Discussion

Numerous studies have shown that Trop2 is highly expressed in multiple epithelial cancers including breast, lung, urothelial, colon, and ovarian cancers^20–25^. Due to the high expression of Trop2 in epithelial cancers, Sacituzumab govitecan, an anti-Trop2-antibody-conjugated to SN-38, has been FDA-approved for triple-negative breast cancer, hormone-positive breast cancer, and urothelial cancer^26–28^. Significant clinical benefits have been observed in clinical trials targeting Trop2 with Sacituzumab govitecan in Triple-negative breast cancer and hormone-positive breast cancer^26,28,29^. In the context of prostate cancer, Trop2 is highly expressed in metastatic castration-resistant prostate cancer and Sacituzumab govitecan is currently tested in clinical trials for patients with metastatic prostate cancer^15,17,30,31^. As we demonstrated that Trop2 is a prognostic tissue biomarker for clinically significant prostate cancer and is associated with worse clinical features, suggesting the great potential of Trop2 targeted therapy as a therapeutic strategy for metastatic prostate cancer.

Clinically significant prostate cancer, which accounts for approximately 15% of all prostate cancer diagnoses, frequently recurs after surgery or radiation therapy and shows high disease-specific mortality^7,32^. Establishing new prognostic biomarkers that can distinguish clinically significant prostate cancer will result in a decrease in overdiagnosis and overtreatment of patients with indolent prostate cancer^9,33^. Several risk stratification systems, such as D'Amico risk groups and the Cancer of the Prostate Risk Assessment (CAPRA) score commonly use clinical T stage, serum prostate-specific antigen (PSA), and biopsy Gleason Score as risk assessment criteria^4,34–36^. However, PSA is not cancer-specific, and the primary biopsy Gleason Score has a more than 20% mismatch compared to the pathologic Gleason Score and the mismatch rate increases to 50% in the second biopsy^37^. Thus, the development of new companion prognostic molecular biomarkers in conjunction with the current risk stratification system will improve the early detection of clinically significant prostate cancer, and provide more accurate risk classification, ultimately guiding precise treatment for multimodal therapies versus active surveillance in prostate cancer patients.

A molecular biomarker with prognostic value is a promising alternative and/or a marker to monitor prostate cancer progression and therapeutic response^38^. The urine-based biomarker can be measured for disease diagnosis and prognosis in a non-invasive manner^39^. In this context and to the best of our knowledge, this is the first study that evaluates shed Trop2 as a potential urine biomarker for clinically significant prostate cancer and a tissue-based predictor for the overall-survival time in prostate cancer. We demonstrate that tissue and urine Trop2 may supplement current clinical biomarkers for the early detection and prediction of clinically significant prostate cancer. We anticipate that patients with elevated urine Trop2, in cooperation with other high-risk criteria, could further facilitate risk stratification and outcome prediction of patients with prostate cancer. Our studies warrant further research in larger cohorts to assess the utility of the urine Trop2 assay as a clinical test and to establish shed Trop2 as a urine biomarker for the early identification of clinically significant prostate cancer and the monitoring of treatment response.

## Methods

All methods were carried out in accordance with the relevant guidelines and regulations of Stanford University and University of California, Los Angeles.

### Immunohistochemistry (IHC)

Trop2 staining was conducted on the Canary Prostate Cancer Tissue Microarray (CPCTA)^19^. Details of the case selection, clinical data, statistical considerations, and TMA construction have been described previously^19^. Briefly, microarrays were constructed using a standardized protocol and included 3 cores of cancer from the largest and highest-grade cancer lesion and a single core of normal peripheral zone prostate tissues from the same patient. Cases were selected at random from available radical prostatectomy specimens performed for localized prostate cancer. All patients had a minimum of 5-years of follow-up and complete clinical and pathological data was collected relevant to prostate cancer including patient age, pre-operative PSA level, clinical stage, pathological stage, Gleason Grade group, follow-up time, and patient status (no evidence of disease, biochemical recurrence, metastases, death from prostate cancer, and overall survival). The resource has been reviewed and approved by the Institution Review Board at Stanford University (#40197).

The TMA slides were deparaffinized at 65 °C for 1 h and incubated with Clearify for 15 min, followed by rehydrated in 100%, 95%, and 70% of ethanol. 10 mM citrate buffer (pH = 6.0) was used for antigen unmasking for 20 min at 95 °C. 3% of hydrogen peroxide was used to block endogenous peroxidase activity. 2.5% horse serum was used for blocking at room temperature for one hour. The primary antibody Goat-anti-Trop2-biotin antibody (R&D Systems; BAF650; 1:50) was incubated, and the slides were kept in a humidified chamber at 4 °C overnight. The slides were washed for 5 min with phosphate-buffered saline (PBS) three times and incubated with a streptavidin horseradish peroxidase (HRP) (Vector Labs; SA-5004; 1:200) for one hour at room temperature. After washing the slides three times with PBS, slides were detected with a DAB kit (Dako). The slides were counterstained with hematoxylin followed by dehydration in ascending ethanol. Slides were mounted and scanned by a NanoZoomer (Hamamatsu) for scoring. Trop2 staining intensity was scored by a genitourinary pathologist blinded to outcomes from 0 to 3 (0 is negative, 1 is weak, 2 is moderate, and 3 is strong as shown in Fig. 1B).

### Statistical analysis

All Trop2 staining was scored by a pathologist with expertise in prostate cancer from the Department of Pathology at Stanford University. The Canary Prostate Cancer Tissue Microarray (CPCTA) cohort contains information on the overall patient survival from the date of radical prostatectomy. Overall survival is the time from radical prostatectomy to death with patients censored at last known alive date if the death date was unknown. The Canary TMA was built substantially around biochemical failure. Cases included samples from men with biochemically recurrent prostate cancer within 5 years of surgery and non-recurrent prostate cancer after 5 years of follow-up. Recurrence was defined as: a single PSA > 0.2 more than 8 weeks after prostatectomy; salvage or secondary therapy; clinical or radiologic evidence of metastasis.

Fisher’s exact test or Wilcoxon test was used to assess the association between Trop2 staining and patient characteristics (seminal vesicle invasion, positive surgical margins, age, pre-operative PSA, Gleason Score, p-stage, extracapsular extension). The Kaplan–Meier (KM) method was used to estimate survival curves in Trop2 positive and Trop2 low/negative patients. Patient characteristics and Trop2 staining were evaluated as predictors of overall survival in both univariate and multivariate Cox proportional hazards models. All tests were two-sided and p-values of 0.05 or less were considered statistically significant. Statistical analysis was carried out using R [R Core Team (2019). R: A language and environment for statistical computing. R Foundation for Statistical Computing, Vienna, Austria. URL https://www.R-project.org/].

### Design and collection of urine samples

We used a case–control design to compare Trop2 urine in prostate cancer going to prostatectomy and non-cancer specimens with long term follow up known not to have ever received a prostate cancer diagnosis to represent the extremes of patient scenarios. After consent (IRB: HSC20050234H), we prospectively and serially enrolled men prior to prostatectomy for our cases. 39 samples from men with clinically significant prostate cancer were used in this study. After the prostatectomy, we requested an unstained representative pathologic slide for cancer and a non-cancer portion of the prostatectomy specimen. For the control group, we used a tissue bank of stored specimens from men who were followed over 10 years with PSA below 1.5 ng/ml and therefore they did not meet clinical criteria for biopsy or significant prostate cancer (n = 40) (IRB HSC20000030H, Supplementary Tables 1, 2). All samples were collected at University of Texas Health Science Center at San Antonio under the approved Institutional Review Board (IRB) protocol (IRB: HSC20050234H). The experimental protocols were approved by University of Texas Health Science Center at San Antonio, Stanford University, and University of California, Los Angeles committee (IRB: HSC20050234H). All experiments were carried out in accordance with the relevant guidelines and regulations of Stanford University and University of California, Los Angeles ethical guidelines and regulations. Informed consent was obtained from all subjects. All methods were carried out in accordance with the relevant guidelines and regulations of Stanford University and University of California, Los Angeles.

### Enzyme-linked immunoassay (ELISA)

Trop2 urine levels were determined by a Sandwich ELISA. Diluted anti-Trop2 capture antibody (SinoBiological, 10428-MM01, 1:100) was coated in a 96-well ELISA plate at 4 °C overnight. The wells were washed three times with PBST (PBS supplied with 0.01% Tween-20). 5% of BSA was used for blocking and incubated at 4 °C overnight. 20 µl of whole urine samples from cancer-free patients and clinically significant prostate cancer patients were incubated for 2 h at room temperature. The plates were washed three times with PBST and then incubated with the Goat-anti-Trop2-biotin detection antibody (R&D Systems; BAF650; 1:250) for 2 h at room temperature. The plates were washed three times with PBST and incubated with a streptavidin-HRP (Thermo Fisher Scientific, PI21134, 1:1000) at room temperature for 30 min. After washing with PBST three times followed by PBS two times, the signals were detected by ultra TMB-ELISA substrate (Thermo Scientific, #34028) for 20 min. After adding the stopping solution (Thermo Fisher Scientific, PIN600), plates were analyzed via plate reader (Promega) at 450 nm. The standard curve for Trop2 concentrations was determined using 0–500 pg/ml recombinant human Trop2 (SinoBiological, 10428-H08H-1).

### Western Blot (WB)

30 µl of the whole urine samples from cancer-free patients and patients with clinically significant prostate cancer were denatured with sodium dodecyl sulfate (SDS) at 95 °C for 5 min. Samples were separated by SDS-PAGE (Invitrogen™ XP08165BOX). The proteins were transferred onto a nitrocellulose membrane (GVS Life Sciences, 1212632). 5% non-fat milk was used for blocking, and the membrane was incubated with a primary antibody (R&D Systems; BAF650; 1:1000) at 4 °C overnight. The HRP-conjugated secondary antibody was incubated (Thermo Fisher Scientific, PI21134, 1:2000) for one hour at room temperature and then developed with ECL Substrate (Thermo Fisher Scientific, 32106).

### Supplementary Information

Supplementary Information.

## Data Availability

The datasets used and analyzed during the current study are available from the corresponding author upon reasonable request.
