# Supplementary material for: High expression of Trop2 is associated with aggressive localized prostate cancer and is a candidate urinary biomarker
Source: Sci Rep. 2024 Jan 4;14:486. doi: 10.1038/s41598-023-50215-z (PMC10766957; doi:10.1038/s41598-023-50215-z)
Supplement: Supplementary file 1 — Supplementary Information. [file 41598_2023_50215_MOESM1_ESM.pdf]

Supplementary Figure 1.

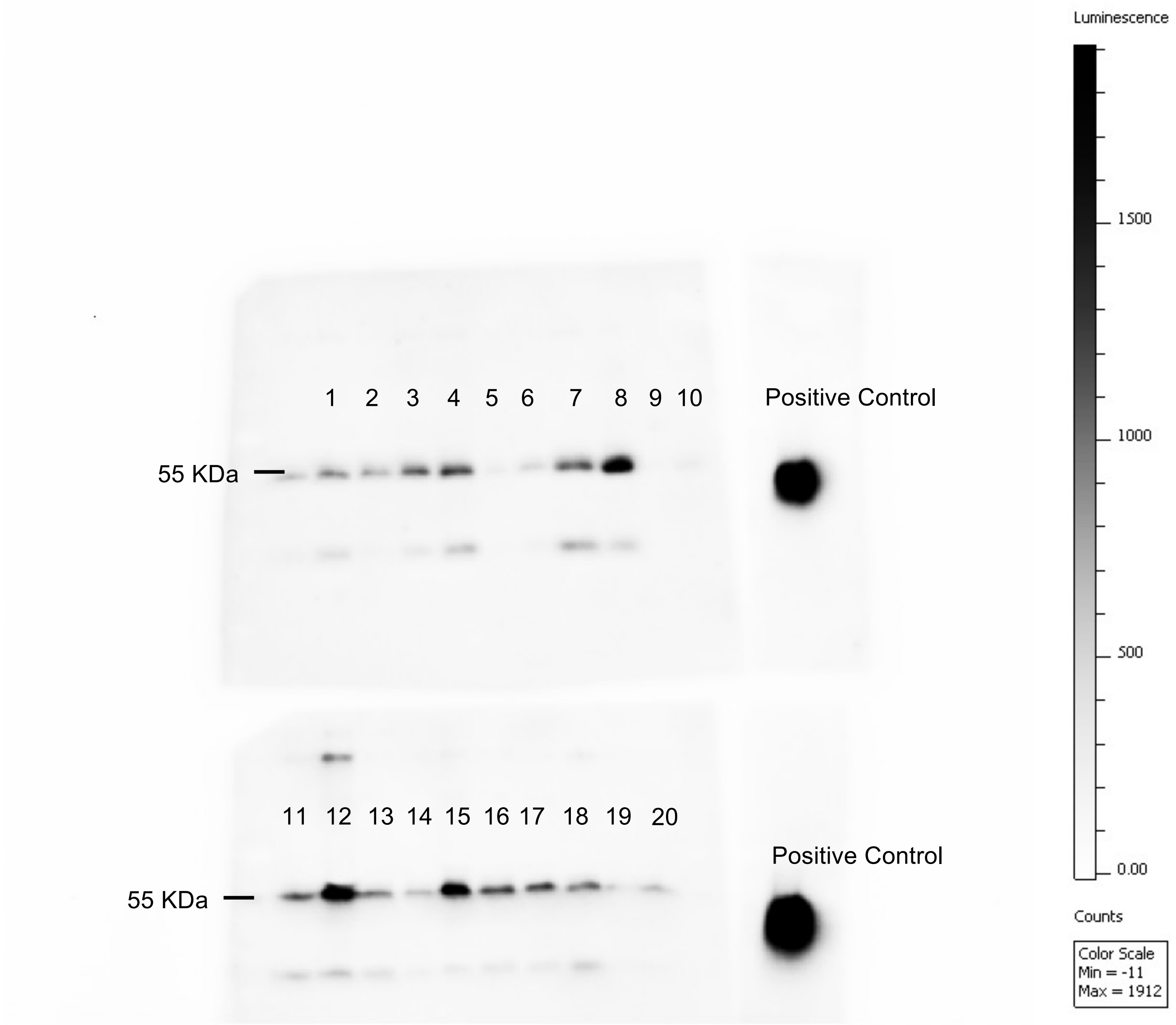

Supplementary Figure. 1 Raw image of Trop2 levels in urine samples from patients with clinically significant prostate cancer from Figure 2C. 10 patients were run in the same gel with positive control, and two blots were developed at the same time with the same exposure time.

Supplementary Table 1. Summary of clinical information of cancer-free patients.

Sample ID	Age at urine collection	Family History Tumor Type	Serum PSA (ng/mL)	Post-DRE Y/N
1	66	Lung	0.5	yes
2	73	Lung	0.4	yes
3	66	Prostate	0.9	yes
4	55	Prostate	0.4	yes
5	55	Breast - Female	0.5	yes
6	64	NA	0.4	yes
7	57	Liver	0.8	yes
8	68	Breast - Female	0.5	yes
9	64	Prostate	0.8	yes
10	63	Leukemia, not otherwise specified	0.8	yes
11	64	Prostate	0.6	yes
12	63	NA	0.3	yes
13	54	NA	1.3	yes
14	61	Leukemia	0.7	yes
15	76	Other	0.5	yes
16	58	NA	0.1	yes
17	60	Prostate	0.6	yes
18	72	NA	0.3	yes
19	65	Lung	0.6	yes
20	64	Prostate	0.1	yes
21	67	NA	0.7	yes
22	57	Prostate	0.3	yes
23	71	NA	0.5	yes
24	56	Prostate	0.3	yes
25	69	Prostate	0.4	yes
26	58	Breast - Female	0.3	yes
27	58	NA	0.9	yes
28	61	NA	0.9	yes
29	50	Breast - Female	0.8	yes
30	47	NA	0.6	yes
31	56	Leukemia, not otherwise specified	0.3	yes
32	56	Other Male Genital	0.5	yes
33	50	Prostate	1.1	yes
34	53	Breast - Female	0.6	yes
35	66	Breast - Female	0.7	yes
36	67	Lung	0.2	yes
37	56	NA	0.5	yes
38	60	NA	0.2	yes
39	53	NA	0.1	yes
40	60	Prostate	0.4	yes

Supplementary Table 2. Summary of clinical information of patients with clinically significant prostate cancer.

ID	Collection	Post DRE	Age	Pre-op PSA	Clinical Grade (ISUP)	Clinical Stage (NCCN)	Pathologic Grade	Pathologic Stage
1	Catheter	N	61	7.94	3	Unfavorable intermediate	4+3	pT2N0
2	Patient void	N	56	6.76	4	High risk	3+4	pT3b
3	Catheter	N	53	6.2	3	Unfavorable intermediate	4+3	pT3a
4	Patient void	N	60	18.1	3	Unfavorable intermediate	4+3	pT3a
5	Catheter	N	61	7	4	High risk	5+4	pT2
6	post DRE void	Y	64	12.46	4	High risk	4+3	pT2NX
7	post DRE void	Y	63	9.67	2	Favorable Intermediate	3+4	pT2
8	Patient void	N	66	5	2	Favorable Intermediate	3+4	pT2a
9	Patient void	N	58	37.2	5	Very High Risk	4+5	pT3
10	Patient void	N	53	5.65	2	Favorable Intermediate	3+4	pT2
11	post DRE void	Y	55	9.84	3	Unfavorable intermediate	3+4	pT2N0
12	Patient void	N	68	12.04	3	Unfavorable intermediate	4+3	pT3aN0
13	post DRE void	Y	64	9	3	Unfavorable intermediate	4+4	pT3a+b
14	patient void	N	66	5.54	3	Unfavorable intermediate	4+3	pT3a
15	Catheter	N	59	7.41	2	Favorable Intermediate	3+4	pT2N0
16	Catheter	N	54	35	4	High risk	4+4	NA
17	post DRE void	Y	65	12.12	2	Unfavorable intermediate	3+4	pT3a/b (left)
18	post DRE void	Y	72	5.6	1	Low risk	3+3	pT3a
19	Catheter	N	56	25.1	3	High risk	3+4	pT2
20	Catheter	N	75	8.39	3	Unfavorable intermediate	4+3	pT2
21	post DRE void	Y	57	3.87	2	Favorable Intermediate	3+4	pT2
22	post DRE void	Y	73	7.35	3	Unfavorable intermediate	4+3	pT3aN0
23	Catheter	N	67	2.87	5	Very High Risk	5+5	pT3a
24	Catheter	N	68	5.91	5	High risk	4+5	pT3b
25	post DRE void	Y	70	4.5	2	Unfavorable intermediate	3+4	pT3
26	post DRE void	Y	63	18.08	5	High risk	5+4	pT3bN1
27	post DRE void	Y	67	10.23	2	Unfavorable intermediate	4+3	pT3bN0
28	Catheter	N	60	7.33	2	Unfavorable intermediate	4+3	pT2N0
29	post DRE void	Y	68	5.94	1	Low risk	4+3	pT2
30	post DRE void	Y	57	26.6	2	High risk	4+4	pT3a
31	Catheter	N	58	7.57	4	High risk	4+5	pT3aN0
32	Catheter	N	54	3.88	2	Favorable Intermediate	3+4	pT2
33	Catheter	N	64	6.1	2	Favorable Intermediate	3+4	pT2
34	Catheter	N	57	5.6	2	Favorable Intermediate	3+4	NA
35	Patient void	N	50	7.11	4	Very High Risk	4+3	pT2
36	Catheter	N	50	5.27	4	High risk	3+4	pT3a
37	Catheter	N	44	6.06	2	Favorable Intermediate	3+4	pT2
38	post DRE void	Y	51	7.99	2	Favorable Intermediate	3+4	NA
39	Catheter	N	65	7.5	3	Unfavorable intermediate	3+4	pT3a
